# Review of Medical Therapies for the Management of Pulmonary Embolism

**DOI:** 10.3390/medicina57020110

**Published:** 2021-01-26

**Authors:** Ladan Panahi, George Udeani, Michael Horseman, Jaye Weston, Nephy Samuel, Merlyn Joseph, Andrea Mora, Daniela Bazan

**Affiliations:** Department of Pharmacy Practice, Texas A&M Rangel College of Pharmacy, 1010 W Ave B, Kingsville, TX 78363, USA; mah0752@tamu.edu (M.H.); jsw1954@tamu.edu (J.W.); nsamuel@tamu.edu (N.S.); joseph@tamu.edu (M.J.); andrea.mora@tamu.edu (A.M.); dbazan@tamu.edu (D.B.)

**Keywords:** pulmonary embolism (PE), venous thromboembolism (VTE), anticoagulants, direct oral anticoagulants (DOAC)

## Abstract

Traditionally, the management of patients with pulmonary embolism has been accomplished with anticoagulant treatment with parenteral heparins and oral vitamin K antagonists. Although the administration of heparins and oral vitamin K antagonists still plays a role in pulmonary embolism management, the use of these therapies are limited due to other options now available. This is due to their toxicity profile, clearance limitations, and many interactions with other medications and nutrients. The emergence of direct oral anticoagulation therapies has led to more options now being available to manage pulmonary embolism in inpatient and outpatient settings conveniently. These oral therapeutic options have opened up opportunities for safe and effective pulmonary embolism management, as more evidence and research is now available about reversal agents and monitoring parameters. The evolution of the pharmacological management of pulmonary embolism has provided us with better understanding regarding the selection of anticoagulants. There is also a better understanding and employment of anticoagulants in pulmonary embolism in special populations, such as patients with liver failure, renal failure, malignancy, and COVID-19.

## 1. Introduction

Pulmonary embolism (PE) is a type of venous thromboembolism (VTE) that is potentially fatal but can be treated with different types of therapy. PE is an obstruction of the pulmonary arteries that can be caused by a clot, tumor, fat, or air. This article focuses on the treatment of blood clots. PE occurs when a portion of a blood clot breaks off and travels until it lodges in the pulmonary arteries [1]. Most deep vein thrombosis will develop in the lower extremities, but up to half can lead to PE [2]. PE is a significant health issue in the US, since there is an increased prevalence of this condition in the elderly. Other risk factors include obesity, heart failure, and cancer [3].

Current anticoagulation management guidelines prefer direct oral anticoagulants (DOAC) for initial and long-term therapy for treating PE [4]. Dabigatran, rivaroxaban, apixaban, and edoxaban are preferred over vitamin K antagonist (VKA) therapy. This is due to the similar risk reduction for recurrent VTE, reduced risk of bleeding, and improved patient and provider convenience over the intensive monitoring associated with VKA therapy [4]. Each of the DOACs has demonstrated similar efficacy outcomes compared to VKA therapy with recurrent embolism [4]. In contrast, the risk of bleeding differs with the DOACs, which demonstrate less risk when compared to warfarin [4]. One possible exception to this, however, is that gastrointestinal bleeding may be higher with dabigatran, rivaroxaban, and edoxaban compared to warfarin. This has been observed in patients treated with atrial fibrillation [4]. Low molecular weight heparin (LMWH) may be considered for treatment as an alternative to DOACs and VKA therapy [4]. These treatment recommendations differ slightly in patients with cancer-associated thrombosis [4]. In such patients, LMWH is still recommended over VKA therapy. Evidence suggesting that LMWH is more effective in reducing recurrent embolism in this population is more reliable in patients who have difficulty tolerating oral intake and removes the need for thorough International Normalized Ratio (INR) monitoring [4].

The duration of anticoagulant therapy for PE is three months, at minimum, which may be extended or indefinite in selected circumstances [4]. In patients with a PE provoked by surgery or a nonsurgical transient risk factor, the recommended duration of anticoagulation is three months [4]. In patients with an unprovoked PE, bleeding risk determines the duration, but in patients with high bleeding risk, the duration remains at three months. In low to moderate bleeding risk, the duration of therapy becomes indefinite [4].

The prevention of PE in hospitalized patients includes either LMWH, low-dose unfractionated heparin (UFH) administered twice or three times daily, or fondaparinux [5]. Bleeding remains a concern with anticoagulation therapy. Reversal agents such as protamine sulfate and vitamin K have been used to manage heparin and warfarin-associated bleeding, respectively. The availability of 4-factor prothrombin complex concentrate (4F-PCC) has resulted in its use over fresh frozen plasma (FFP) as a reversal agent for warfarin-associated bleeding. The reversal agent, andexanetandexanet alfa, has become available for apixaban and rivaroxaban-associated bleeding, while the monoclonal antibody idarucizumab has been developed and employed as a reversal agent for dabigatran. The availability of these agents has allowed clinicians to push the boundaries of PE management with confidence in both the inpatient and outpatient settings.

PE in special populations presents a challenge, particularly in the modulation of therapies such as DOACs, LMWH, UFH, and VKA in obesity, renal dysfunction, liver impairment, cancer, and COVID-19 patients.

## 2. Unfractionated Heparin (UFH)

UFH is derived from porcine or bovine tissue. It is a parenteral anticoagulant that works by inactivating thrombin (IIa) and factor Xa via antithrombin. Dosing and special considerations for UFH are discussed in Table 1. The short half-life ranges from 0.5 to 1.5 h, which makes UFH the anticoagulant of choice in patients with PE. This is particularly the case in patients with high bleeding risk, critical illness, or who are in need of a surgical/invasive procedure. The anticoagulant effect occurs rapidly and dissipates within hours of IV discontinuation [6]. Another advantage to UFH is its unique metabolism and clearance through the reticuloendothelial system, which make it a desirable option for patients with poor and/or unstable renal function (creatinine clearance (CrCL) < 30 mL/min) [1]. Since UFH lacks cytochrome P450 enzyme activity in the liver, drug interactions are predominantly limited to increased bleeding risk with concurrent anticoagulant and antiplatelet therapy. The IV route is the preferred mode of administration in shock and/or hypotension due to the absorption variability from subcutaneous tissues secondary to UFH plasma protein binding [6].

Heparin-associated thrombocytopenia can present in two forms. There is an early, benign, reversible nonimmune thrombocytopenia form and a late, more serious immunoglobulin G (IgG)-mediated immune thrombocytopenia type, referred to as heparin-induced thrombocytopenia (HIT). HIT poses a concern with UFH use in the treatment of PE, with the overall incidence being reported to be up to 7% in patients with a mortality of 20% to 30% and varies depending on factors such as patient population (surgical vs. medicine), duration of heparin use, and the type of heparin administered [7,8]. UFH has a three-fold higher risk of HIT compared to LMWH and serious limb-threatening and life-threatening complications [9]. The mechanism of HIT stems from IgG formation against the heparin/PF4 complex on platelets. Once IgG binds to the heparin/PF4 complex on platelets, the platelets become activated, resulting in venous and arterial thrombi formation [9,10]. Monitoring platelets every 2 to 4 days or more frequently in higher-risk patients is recommended in patients on treatment doses of heparin [10].

Some adverse drug reactions of concern are thrombocytopenia and major bleeding, such as intracranial and gastrointestinal bleeds. Another side effect of concern is the significant reduction in bone density reported in about 30% of adult patients and the symptomatic bone fractures that occur in 2% to 3% of adult patients receiving heparin for at least 1 month or more [11].

## 3. Low Molecular Weight Heparins (LMWH)

LMWHs, including enoxaparin and dalteparin, are defined as having a mean molecular weight that is less than 50% of that of UFH. They offer the advantage of consistent anticoagulant effect administered subcutaneously and dosing by body weight. LMWHs, including enoxaparin and dalteparin, are defined as having a mean molecular weight that is less than 50% of that of UFH. They offer the advantage of consistent anticoagulant effect administered subcutaneously and dosing by body weight.

LMWH is currently the preferred anticoagulant for active malignancy and pregnancy. However, newer study findings support the notion that DOACs may benefit VTE treatment in cancer patients [13]. Routine anti-Xa monitoring is generally not recommended for enoxaparin but can be considered in patients with severe or unstable renal function and obese patients with a BMI ≥ 40 kg/m^2^ (or >190 kg) who will be on enoxaparin for longer than 1 to 2 weeks [12,13]. The anti-Xa level peak is drawn four hours after the enoxaparin dose is administered when a steady state is achieved with a target range of 0.6 to 1.0 IU/mL [13,21]. Monitoring anti-Xa activity in morbidly obese patients > 120 to 140 kg and patients with severe or unstable renal function may be considered for dalteparin with a target anti-Xa range of 0.5 to 1.5 IU/mL [14]. Similarly, to enoxaparin, the anti-Xa levels in patients taking dalteparin must be drawn 4 to 6 h after the dalteparin dose is administered and once a steady state is achieved (receiving at least 3 to 4 doses) [14].

While the risk of HIT is lower with LMWH in comparison to UFH, a baseline platelet count is suggested as a basis from which to contemplate the development of HIT. Further treatment with LMWH should be avoided in patients with a known history of HIT. Although it less likely to trigger the formation of HIT antibodies than UFH, LMWHs are just as effective as UFH in triggering platelet activation by HIT antibodies [11].

### 3.1. Vitamin K Antagonists

VKAs such as warfarin continue to play a role in PE treatment, particularly in patients with severe renal insufficiency, antiphospholipid syndrome, and financial constraints who are unable to afford DOACs [22]. International Normalized Ratio (INR) monitoring is recommended for warfarin monitoring. Although initially an INR increase may be observed, patients might be in a hypercoagulable state during the first few days of warfarin therapy due to the drug’s long half-life, slow depletion of factor II, and rapid depletion of anticoagulant protein C [23]. Due to this initial hypercoagulable state, bridging with UFH, LMWH, or fondaparinux for at least 5 days and until the INR reaches the therapeutic range of 2 to 3 is recommended [24]. Foods high in vitamin K such as oils, fats, liver, nuts, and green vegetables may interact and limit the efficacy of warfarin. Patients are educated about maintaining consistency in their food intake and discussing any dietary changes with their healthcare provider prior to making any changes. Due to the increased potential for significant interactions with medications that are inducers and inhibitors of CYP2C9, 2C19, 1A2, and 3A4, patients must be counseled on notifying their healthcare provider about medication changes prior to the change so that their healthcare provider can devise a dosing and monitoring plan to ensure that the warfarin is not affected and that their INR remains in the therapeutic range [25]. Not only does the INR monitoring pose a limitation for warfarin use, but its vast food and drug interactions also limit the desire to initiate warfarin for long-term therapy.

### 3.2. Factor Xa Inhibitor Injectable

Fondaparinux (Arixtra^®^) is a synthetic pentasaccharide anticoagulant selectively inhibiting factor Xa via antithrombin-dependent actions with no inhibition of thrombin (factor IIa) [26]. Unlike LMWH and UFH, fondaparinux inhibits a targeted step in the coagulation cascade that leads to its anticoagulant effect [26]. Fondaparinux is currently approved in the United States for the treatment of PE in conjunction with warfarin [26]. Fondaparinux does not require laboratory monitoring and lacks a specific reversal agent.

## 4. DOACs

Apixaban is an oral direct factor Xa inhibitor approved for stroke risk reduction and deep venous thrombosis (DVT) prophylaxis, which may lead to PE following knee and hip replacement surgery, and DVT and PE treatment [15]. New uses include antithrombotic therapy after acute coronary syndrome (ACS) or percutaneous coronary intervention (PCI) in atrial fibrillation and HIT [22,23,24]. The oral bioavailability is approximately 50%, with most of the drug absorption occurring in the small intestine [27]. Drug elimination occurring via the metabolism through the CYP3A4 systems in the intestine and liver and the P-glycoprotein system can be enhanced through drug–drug interactions [22,25,26,27,28,29]. Once absorbed, the terminal half-life ranges between 8 and 12 h, with steady state achieved within three days [22,25,26,27,28,29]. The premature discontinuation of any anticoagulant, including apixaban, increases the risk of thrombotic events and is listed as a black box warning [15]. Epidural or spinal hematomas may occur in patients treated with apixaban in neuraxial anesthesia or spinal puncture. These hematomas have long-term consequences, such as permanent paralysis. Such risks should be taken into account when patients on apixaban therapy are scheduled for spinal procedures [15].

Rivaroxaban is a direct factor Xa inhibitor with an oral bioavailability that ranges from approximately 80% to 100% for a 10 mg dose and up to 66% for a 20 mg [30,31,32]. Doses ≥15 mg are improved with food [30,31,32]. Rivaroxaban’s terminal half-life ranges between 5 and 9 h, with a prolonged half-life of 11–13 h seen in elderly patients [30,31,32]. The steady-state concentrations for this agent tend to occur within three days. Renal elimination accounts for approximately 36% of unchanged drug, its use in patients with a CrCL < 30 mL/min is not advised, and <15 mL/min employment is contraindicated [30,31,32]. Rivaroxaban is not dialyzable [30,31,32]. Rivaroxaban follows a similar elimination pattern to that of apixaban, with metabolism through the CYP3A4 systems accounting for 57% elimination [30,31,32]. P-glycoprotein system inhibitors may lead to elevated rivaroxaban serum levels [27,29,33]. The premature discontinuation of any anticoagulant, including rivaroxaban, increases the risk of thrombotic events and is listed as a black box warning [16]. To minimize this risk, an alternate coverage should be considered, should rivaroxaban be discontinued for a reason apart from pathological bleeding or therapy completion [16]. Epidural or spinal hematomas have been observed in patients managed with rivaroxaban undergoing neuraxial anesthesia or spinal puncture. Such hematomas are known to result in long-term or permanent paralysis [16].

Dabigatran (Pradaxa^®^) is a DOAC that works as a direct thrombin (IIa) inhibitor. The dose for PE treatment is 150 mg orally twice a day after 5 to 10 days of parenteral anticoagulation in patients with adequate renal function (CrCl > 30 mL/min) [20]. Although rivaroxaban and apixaban do not require bridging, dabigatran needs bridging with UFH or LMWH for 5 to 10 days [1]. Dabigatran should be initiated 0 to 2 h before the next dose of parenteral anticoagulant would have been due, or at the time of discontinuation of UFH continuous infusion [20]. Similarly to all anticoagulants, any medication with the potential to increase bleeding risk, such as antiplatelets, anticoagulants, and thrombolytics, will be considered a major drug interaction when administered concomitantly with dabigatran [20]. P-glycoprotein (P-gp) inducers, such as phenobarbital, rifampin, and fosphenytoin, reduce exposure to dabigatran and should be avoided [20]. In contrast, P-glycoprotein (P-gp) inhibitors increase exposure to dabigatran, and recommendations vary based on the P-gp inhibitor and the indication for dabigatran use [20].

Renal impairment and P-gp inhibition are the major independent risk factors for increased dabigatran exposure and increased risk of bleeding. Renal function assessment is recommended before the initiation of dabigatran therapy and clinical situations that may be associated with a decline in renal function. Dose adjustments for dabigatran doses in patients with severe renal impairment (CrCl 30 mL/min or less) are recommended. Caution must be used in the elderly, as the risk of stroke and bleeding increases with age, as seen in an analysis of the RE-LY (Randomized Evaluation of Long Term Anticoagulant Therapy) trial [34]. Dabigatran is contraindicated for use in patients with mechanical heart valves. The RE-ALIGN (Randomized, Phase II Study to Evaluate the sAfety and Pharmacokinetics of oraL dabIGatran Etexilate in Patients after Heart Valve replacement) trial was terminated early due to thromboembolic events (valve thrombosis, stroke, and myocardial infarction), and major bleeding was observed in the dabigatran group compared to the warfarin group in heart valve patients [35].

Besides major and minor bleeding, gastrointestinal adverse effects have been reported in studies at an incidence rate ranging from 24.7% to 35% [20]. The gastrointestinal adverse effects reported include but are not limited to dyspepsia, gastritis, abdominal pain or discomfort, and epigastric discomfort [20]. Due to the prevalence of these adverse effects, it is necessary to educate the patient about the potential side effects and advise them not to abruptly discontinue the medication before notifying their healthcare provider [20].

One distinguishing feature of dabigatran is the increase in bioavailability by 75% that occurs if the capsule is broken, chewed, or emptied out of the capsule shell [20]. This leads to an increased risk of toxicity, such as major bleeds [20]. Since the capsule cannot be manipulated due to the stated reason, the medication should not be administered via a feeding tube of any type [20]. The medication must be kept in its original container. It must be discarded after four months from the date the package was opened due to the lack of stability with light or humidity exposure that may lead to product breakdown and potency loss [20].

Edoxaban, an oral direct factor-X inhibitor, was approved in the United States and Europe in 2015 for the treatment of PE. Approval was based primarily on the Hokusai VTE study, which evaluated 3319 patients with PE. The trial showed that edoxaban was not inferior to warfarin but had a lower bleeding risk [36]. Edoxaban is only approved for prophylaxis to reduce the risk of stroke and systemic embolism (SE) for patients with nonvalvular atrial fibrillation and for the treatment of VTE (including PE) [37]. In contrast to apixaban and rivaroxaban, it does not have indications for the prophylaxis of recurrent VTE or for the prophylaxis of the total knee and hip arthroplasty [38].

## 5. Monitoring Parameters for Anticoagulation Therapy

Therapeutic anticoagulation is the gold standard for the management of VTE. The narrow therapeutic efficacy window creates challenges in drug selection and monitoring to deliver the appropriate dose to prevent further embolic events while not causing a life-threatening bleed. Historically, guidelines in VTE management include the option of continuous UFH; however, LMWH and direct factor Xa inhibitors have begun to displace UFH as first-line agents [1,5,20]. Traditionally, aPTT has served as the marker of therapeutic anticoagulation in patients with VTE due to the exclusive use of UFH [39,40,41,42,43,44,45,46]. Despite decades of experience with aPTT values, challenges in the precision monitoring of anticoagulation continue to create therapeutic dilemmas for clinicians [39,47]. The lack of standardized methods in monitoring and individualized patient factors, including biological variables and heparin resistance, are credited as most problematic when using aPTT to measure the effect of UFH [40,41]. While aPTT values remain the standard for measuring the effects of UFH, anti-factor Xa (anti-Xa) heparin assay (HA) is recommended as a monitoring option in place of aPTT values by the American College of Chest Physicians (ACCP) and the College of American Pathologists (CAP) [20,38]. Anti-Xa levels demonstrate greater consistency in measuring anticoagulation, since they are based on the functional activity of all heparins [42,43]. Comparatively, the aPTT test measures the function of the intrinsic and common pathways of the coagulation cascade, which can have significant variability amongst individuals [37,39,40].

The implementation of heparin dosing protocols has improved the uniformity of therapeutic anticoagulation with UFH. The majority of these protocols are “weight-based”, and changes in the units of UFH per hour are adjusted based on the aPTT values [44]. This method of dosing UFH has led to concerns regarding both “over” and “under” anticoagulation using aPTT-level directed therapy. Combining aPTT and anti-Xa levels has been proposed as a method to overcome the variable swings commonly seen in patients with multiple comorbidities, obesity, older age (>70 yrs), 16 drug-induced coagulopathies, pharmacokinetic changes, and preexisting genetic alterations [37,38,39,41,44,45,46]. Some hospital systems have transitioned from the use of aPTT to anti-Xa HA altogether and are reporting the faster attainment of therapeutic anticoagulation in addition to the elimination of multiple laboratory tests and dosage changes [42].

Based on numerous studies, anti-Xa levels have been shown to be a viable alternative to aPTT in an attempt to achieve and maintain therapeutic anticoagulant levels with UFH [35,40,44,45,46,48]. Anti-Xa levels have provided a more consistent method of monitoring a patient’s response to UFH and demonstrate fewer blood samples and dosage adjustment compared to aPTT values [39,43].

INR monitoring is recommended more frequently upon warfarin initiation (once a day) and can be extended to typically once a month once INR is stable and in a therapeutic range [25].

Routine laboratory monitoring is not indicated for DOAC, but anti-factor Xa (FXa) can be useful for excluding clinically important levels of DOAC [49]. Edoxaban may elevate the PT and aPTT, but FXa activity may be a better measure of effectiveness [38]. Fondaparinux does not require laboratory monitoring as well. Anti-factor Xa activity is probably the assay for monitoring fondaparinux but is not usually obtained in real time [50]. For the qualitative assessment of dabigatran, thrombin time (TT) and aPTT may be used, with TT being sensitive to dabigatran even at low drug concentrations [49].

## 6. Reversal Agents

As with all of the anticoagulants available in the market, bleeding is the main side effect of concern, particularly major life-threatening bleeds such as intracranial and gastrointestinal bleeds. As a result of the increased bleeding risk associated with anticoagulants, the desire to obtain a reversal agent specific to a given anticoagulant is essential (Table 2). Protamine sulfate is a reversal agent indicated for the reversal of UFH- and LMWH-associated bleeds. This agent is administered by a slow IV infusion at doses < 5 mg/min due to the concerns of anaphylaxis, hypotension, bradycardia, and respiratory toxicity associated with the rapid infusion [12]. Due to UFH’s short half-life, a reversal agent may not be necessary for a majority of cases, as the UFH effect will normalize due to its rapid clearance. LMWHs are only partially reversed by protamine (60 to 80%), since binding only occurs with long, large molecular weight heparin proteins [12]. Due to its small size, fondaparinux is not reversed by protamine [12]. Protamine is known to interact with platelets, fibrinogen, and other plasma proteins. This may result in an anticoagulant effect of its own; thus, the minimal amount of protamine required to neutralize heparin present in the plasma should be administered.

Vitamin K is recommended for warfarin if the INR > 10 without significant bleeding and with repeated vitamin K doses every 6 to 24 h as needed [51]. Vitamin K can be administered by IV or the oral route, with a preference for oral administration; there is an associated risk of anaphylactoid reactions in 3 out of 10,000 patients when IV formulations were administered [52]. Subcutaneous injection is not recommended due to a delayed and unpredictable effect [51]. The availability of 4F-PCC (KCentra^®^) during the last decade has resulted in its use over FFP as a reversal agent for warfarin-associated bleeding complications. This agent has been employed especially for life-threatening bleeding, where 4F-PCC has demonstrated 25 times more potency in replacing vitamin K-dependent clotting factors than FFP [49]. The limitations of FFP in comparison to 4F-PCC include its slower onset, risks of allergic reaction and infection transmission, blood group compatibility, longer preparation time, and higher volume [49]. A major advantage of 4F-PCCs is its ability to be stored at room temperature as a lyophilized powder and the fact that it can quickly be reconstituted and administered [49]. With life-threatening bleeding, the addition of vitamin K 5 to 10 mg by slow IV infusion is suggested [53]. There is a specific reversal agent approved for dabigatran. The agent named idarucizumab (Praxbind^®^) is a monoclonal antibody with 350 times more affinity for dabigatran than thrombin [14,54]. Diuresis may also help with excretion of dabigatran as well [20].

Andexanet alfa (Andexxa^®^) can be used to reverse apixaban, rivaroxaban, and edoxaban (off label) in life-threatening or uncontrollable bleeding. The dosing is based on the specific factor Xa agent-inhibitor to be reversed, dose, and the time since the last dose was administered [55]. Andexanet alfa 400 mg intravenous bolus is administered at a rate of 30 mg/min, followed by 4 mg/min via continuous infusion for up to 120 min, to reverse apixaban (5 mg or less) or rivaroxaban (10 mg or less), administered within 8 h or if the time is unknown [55]. Rivaroxaban doses of greater than 10 mg, or if an unknown amount is administered within 8 h or at an unknown time, is managed with the high dose andexanetandexanet alfa regimen. High-dose andexanetandexanet alfa is also indicated for apixaban doses of greater than 5 mg, or, if unknown, administered within 8 h or an unknown time. The high-dose andexanetandexanet alfa regimen is 800 mg intravenous bolus at a target rate of 30 mg/min, followed by 8 mg/min continuous infusion for up to 120 min [55]. The safety and efficacy of administering more than one dose of any of these regimens have not been established [55].

In the absence of either idarucizumab or andexanet alfa for DOAC reversal, administering prothrombin complex concentrate (PCC) or activated prothrombin complex concentrate (aPCC) are alternatives to consider. This is based on the limited available human and animal in vitro studies [49]. Activated charcoal for the known recent ingestion of DOACs may also be effective when DOAC ingestion occurs within the last 2 to 4 h [49].

For Fondaparinux, recombinant activated factor VII and aPCC have some data in human and animal studies, respectively, as reversal agents. Both andexanet alfa (a recombinant factor Xa) and aripazine have been shown to bind to Xa inhibitors but lack any human data with fondaparinux [50].

## 7. Thrombolytic Therapy

Thrombolytic therapy for PE may be administered systemically or directed by a catheter into the pulmonary arteries to accelerate the resolution of acute PE. Thrombolytic therapy can lower pulmonary artery pressure and increase arterial oxygenation [22]. Studies limiting thrombotic therapy in acute PE have shown the best outcomes by restricting it to patients presenting with a massive (high risk) PE [22,56]. Patients presenting with hemodynamic instability, right ventricular dysfunction, and without significant risk of bleeding are considered potential candidates for emergency thrombolytic therapy [4,57]. The results from timely administration of thrombolytic therapy may be seen within 36 h [58]. Mortality rates occur in up to 30% of patients categorized as high risk and make the timing of therapeutic intervention critical [59,60,61]. Adverse events, especially the high incidence of bleeding and hemorrhagic stroke, require careful consideration prior to starting thrombolytic therapy [62,63]. Systemic alteplase (Activase^®^) is the only FDA-approved thrombolytic for the management of acute massive (high risk) hemodynamically unstable PE [64]. All anticoagulants must be stopped prior to the initiation of alteplase and 100 mg IV infused over 2 h is the most common regimen. Alternative weight-based regimens for patients weighing <65 kg may be considered [65]. A weight-based regimen with a 15 mg bolus followed by 0.75 mg/kg over 30 min (max 50 mg) and then 0.5 mg/kg over the next 30 min (max 35 mg) has shown efficacy without increased bleeding [65]. Intra-catheter directed alteplase 0.5–2 mg/h for 2–15 h for a total dose of 4–24 mg has been used successfully in facilities by experienced physicians and well-designed protocols, but this route of administration is not FDA approved [66]. Tenecteplase (TNKase^®^) and reteplase (Retavase^®^) have been used for acute massive (high risk) PE, but are not FDA approved for use in patients with PE [67,68].

## 8. Special Population: Obesity

The anticoagulant dosing of obese patients with PE or other indications has not been well studied [69]. The bulk of the data come from pharmacokinetic/pharmacodynamic (PK/PD) studies and subgroup analyses of premarketing trials comparing obese patients to patients with normal body weight [56,57,58,70]. Most of the current information appears more limited with respect to DOACs. The data for warfarin are more robust, although the usage of this agent appears to be on the decline [69].

Warfarin pharmacokinetics have been compared in various studies of obese patients with a BMI > 30 and 40 kg/m^2^ to normal body weight subjects. These studies found that obesity was associated with a greater delay and dosage to achieve a therapeutic INR. Data associated with the risk of bleeding, however, have been conflicting, indicating that obesity may or may not increase the risk [69].

Dabigatran is a direct thrombin inhibitor that sets it apart from other DOACs. Subgroup analyses of Phase 3 trials for VTE (RE-LY) and atrial fibrillation (RECOVER) suggested no significant differences in the efficacy and safety outcomes of obese patients in comparison to those with lower body weights [49,50]. In contrast, treatment failures have been documented in morbidly obese patients using standard doses. The authors reported that standard doses failed to achieve “therapeutic levels” suggesting a higher volume of distribution (Vd) and a higher clearance [51,52].

The factor ten inhibitors, rivaroxaban, apixaban, and edoxaban, also have some data in obese populations. Rivaroxaban was evaluated in a small PK/PD study involving a heterogeneous group of subjects including those in the obese range (>120 kg). The half-life, Vd, and clearance declined slightly with increasing body weight. The authors felt the declines were not clinically relevant [53]. A separate PK/PD analysis using pooled data from the phase ll EINSTEIN DVT and ODIXa-DVT trials reported similar findings. These studies enrolled some patients with BMIs > 35 kg/m^2^ and found clinical benefits comparable across all weight groups [55]. The authors of this analysis also concluded that standard doses should be sufficient to treat obese patients; however, a review questioned whether the data were robust enough to draw that conclusion [55,71].

In the apixaban’s phase 1 PK/PD study, the pharmacokinetic data were somewhat different compared to those for rivaroxaban. The overall Vd was higher in subjects > 120 kg or BMI > 30 kg/m^2^ than normal body weight subjects. The half-life declined almost proportionately to the increase in Vd (27% ↓ versus 24% ↑ for half-life and Vd respectively). Peak level and area under the plasma drug concentration-time curve (AUC) were also reduced in the higher body weight subjects. The authors concluded the differences were unlikely to be of clinical significance [56]. The phase 3 trials, ARISTOTLE and AMPLIFY, contained a significant proportion of patients with body weights >100 kg and BMIs above 30 kg/m^2^. Subgroup analyses found no differences in efficacy; however, more bleeding episodes were reported in the ARISTOTLE trial. Whether the increase in hemorrhagic complications between these trials was due to age, renal clearance, or other patient-specific factors is unclear [57,58,59].

Edoxaban has less data than other DOACs. Phase 3 Hokusai-VTE enrolled a large group of patients >100 kg. There was no difference in efficacy and safety compared to groups with other weights [60].

A recent international retrospective study of LMWHs examined dosing in obesity with regard to capped (<18,000 IU/d) and uncapped dosages (>18,000 IU/d). The data were obtained from the RIETE Registry, a large prospective case series of patients with VTE. LMWHs included enoxaparin, dalteparin, and tinzaparin. The authors reported that the results may be subject to selection bias despite attempts to control for potential confounders in multivariable analysis. Nevertheless, they found that after adjustment for multiple potential confounders, patients with obesity (>100 kg or BMI > 30 kg/m^2^) who received capped doses were at a lower risk of having the composite outcome of VTE recurrences, major bleeding, or all-cause death at 15 and 90 days. Bleeding was also reduced with capped dosages [61].

A recent retrospective study of UFH for acute venous thromboembolism (VTE) compared three body mass index (BMI) cohorts: (i) non-obese (less than 30 kg/m^2^), (ii) obese (30 to 39.9 kg/m^2^), and (iii) morbid obesity (≥40 kg/m^2^). Dosing employed, was based on actual body weight. The median times to therapeutic aPTT were reported as 16.4, 16.6, and 17.1 h in each of the three cohorts [62].

Obese patients on warfarin may require a higher dose or more time to achieve a therapeutic INR. Bleeding risk may or may not be greater. Available data for DOACs other than dabigatran suggested usual doses may not negatively impact efficacy in obese patients. Apixaban studies reported conflicting results on bleeding risk. However, there is probably insufficient data for DOACs to suggest that usual doses would be adequate in the subgroup of morbidly obese patients. Obese patients with capped doses of LMWHs may have better efficacy and safety outcomes. Capped dosages are conditionally recommended in the 2018 American Society of Hematology guidelines [63].

## 9. Special Population: Renal Dysfunction

Anticoagulants have been evaluated in CKD and ESKD patients in various PK/PD and clinical efficacy trials [64]. The PK/PD of warfarin in CKD and ESKD is not completely understood [64,65]. Official dosing guidelines do not recommend an alteration of dose [64]. Warfarin is extensively metabolized by the cytochrome P450 type 2C9 (CYP2C9) enzyme [72]. Although not removed by dialysis, there are no data evaluating whether this procedure alters its pharmacokinetics and pharmacodynamics [64]. One study comparing individuals with a GFR of 30–59 mL/min and healthy controls reported a shorter half-life and increased clearance. Other data showed that CKD, especially GFRs < 30 mL/min per 1.73 m^2^ or ESKD, complicates warfarin therapy [67,68]. These data reported that lower doses were required to maintain therapeutic INR with greater fluctuations in INR values and higher risks of major bleeding events for any given INR value. A recent meta-analysis of ESKD patients with atrial fibrillation found that warfarin had no benefit in reducing ischemic stroke incidence. The authors concluded that the drug appeared to be associated with a significantly higher risk of hemorrhagic stroke but no increased risk of other types of major bleeding. They also found no change in mortality [69,73]. How all these data apply to other indications such as PE is unknown.

The various DOACS have also been evaluated in renal disease. A small study of dabigatran using reduced doses (150 mg daily for CKD and 50 mg daily for ESKD) examined the effect of CKD and ESKD on pharmacokinetic parameters. Subjects with a creatinine clearance <30 mls/min demonstrated a 6.5-time increase in the AUC with a doubling of the half-life compared to normal controls [74].

Rivaroxaban has somewhat conflicting data in CKD and ESKD. In a phase 3 trial subgroup analysis of the Rivaroxaban Once Daily Oral Direct Factor Xa Inhibition Compared with Vitamin K Antagonism for Prevention of Stroke and Embolism Trial in Atrial Fibrillation (ROCKET AF), the authors reported that reduced renal function (creatinine clearances < 80 mL/min) had no impact on rivaroxaban’s effectiveness and safety [75]. In contrast, a PK/PD study comparing dialysis patients to normal controls reported a 56% increase in AUC when 15 mg doses were administered after dialysis [76]. The AUC was decreased by only 5% when administered pre-dialysis suggesting dialysis is ineffective in clearing the drug [76]. Another PK/PD study comparing three ranges of renal insufficiency to normal controls reported AUCs of 1.4-, 1.5-, and 1.6-fold higher in cases of creatinine clearance concentrations of 50–80, 30–50, and <30 mL/min, respectively [70].

Pharmacokinetic data for apixaban appear similar to those of rivaroxaban, except that the incremental upsurges in AUC are somewhat lower in magnitude. A small PK/PD study using a 5 mg dose in ESKD patients and normal controls found a 36% increase in AUC in the ESKD group compared to controls. The apixaban dose was administered pre-dialysis [77]. Another small PK/PD study used a single 10 mg dose and compared subjects with varying degrees of CKD to normal controls [12]. Compare to the controls, the AUCs increased by 16%, 29%, and 38% in the CKD cohorts with creatinine clearances of 50–80, 30–50, and <30 mL/min, respectively. The mean half-life was only slightly increased in the total CKD population (17 h) compared to the controls (15 h) [77]. The overall results for both studies were not unexpected, as apixaban is metabolized more extensively and demonstrates less unchanged renal elimination than other DOACs [1]. Again, the drug appears to be poorly dialyzable [64].

Edoxaban was evaluated in a small PK/PD study comparing CKD subjects to normal controls. The data, available as an abstract only, showed that the mean AUCs increased by 32%, 74%, and 72% with creatinine clearances of 50–80, 30–50, and <30 mL/min (not on dialysis), respectively [64]. The mean AUC for subjects on peritoneal dialysis was 93% [64]. Similar to other Factor Xa inhibitors, edoxaban is poorly cleared by hemodialysis [78].

Probably the best data for LMWH come from a subgroup analysis of the ExTRACT TIMI 25 trial. This study compared a 1 mg/kg per day dose of enoxaparin to UFH in ST- elevation myocardial infarction (STEMI) patients with CrCLs less than 30 mL. There was no significant difference in mortality or recurrent MI (33 versus 37.7%) and in bleeding risk (5.7 versus 2.8%) for enoxaparin and UFH, respectively. Mortality increased for both drugs as renal function declined [79].

UFH is rapidly eliminated by the reticular endothelial system and, to a lesser extent, the kidney. However, the degree to which the RES functions in this role varies from person to person. Nevertheless, lower doses in VTE treatment are recommended to reduce bleeding risk. An advantage for UFH over enoxaparin is the ability to reverse the former easier with protamine [15,80].

All DOACs increase AUC with the magnitude inversely proportional to renal function. These drugs may be the anticoagulants of choice for stage 1, 2, and 3 CKD [81]. Some sources recommend apixaban as an alternative to warfarin in selected patients with ESKD [64]. Warfarin, although predominately metabolized in the liver, may a require lower dose to reach a therapeutic INR. Historically, it is the oral anticoagulant of choice in ESKD [15,80]. However, how does warfarin’s potential lack of benefit in ESKD patients with AF apply to PE? Are LMWHs better than UFH? Both were associated with similar mortality increases in STEMI patients with declining kidney function. Should these data also apply to renal failure patients and PE?

## 10. Special Population: Liver Dysfunction

Patients with cirrhosis typically have elevated INR and aPTT. As a result, there have been misconceptions that these patients are “autoanticoagulated”. However, patients with liver disease may have a substantially increased risk of VTE (RR 1.74 (95% CI, 1.54–1.95) for liver cirrhosis; 1.87 (95% CI, 1.73–2.03) for non-cirrhotic liver disease) [81]. A meta-analysis in patients with liver disease found the incidence of PE from nine studies to be 0.28% (95% CI 0.13–0.49%) and the prevalence of PE from two studies to be 0.36% (95% CI 0.13–0.7%) [82].

Determining the ideal anticoagulant to use after a PE in a patient with liver cirrhosis may be challenging. Warfarin can be safely used in patients with liver dysfunction. Elevations in baseline INR due to liver disease however may lead to unclear INR targets during warfarin therapy [83,84]. LMWHs have a good safety profile with liver dysfunction; however, subcutaneous administration may limit compliance, and lower anti-Xa levels in liver dysfunction limits efficacy monitoring [85,86]. Finally, there is a lack of data on using DOACs in patients with liver disease. Clinical trials with DOACs excluded patients with liver disease as most DOACs are predominantly cleared hepatically (apixaban 75%, rivaroxaban 65%, edoxaban 50%, and dabigatran 20%) [72,87]. Therefore, dosing recommendations are derived from pharmacokinetic studies (Table 3).

Regardless of which anticoagulant is chosen, the risk of bleeding should be thoroughly evaluated. In patients with liver disease, bleeding can be due to varices, portal hypertensive gastropathy, peptic ulcer disease, and arteriovenous malformations. A small, 45-patient cohort study comparing DOACs to warfarin/LMWH found no difference in thrombotic events. Still, there were significantly fewer major bleeding episodes in the DOAC group (1 patient [4%] vs. 5 patients [28%], *p* = 0.03) [88].

## 11. Special Population: Cancer Patients

Cancer patients have a five to seven-fold increased risk for VTE within the first year of diagnosis [89]. Additionally, VTE is considered an independent predictor of mortality in these patients [90,91]. The pathophysiological and epidemiological association between PE and cancer is well established. For decades, LMWHs were considered a first-line therapy for cancer-associated PEs, as knowledge of the efficacy and safety of DOACs in cancer patients was lacking [76,92,93]. Since then, four large randomized control trials have been published comparing DOACs with LMWH which have highlighted the utilization of most DOACs for the treatment of PEs [76,79,94,95].

The Hokusai VTE Cancer trial randomized 1050 cancer patients with acute VTE to either edoxaban, an oral direct factor Xa inhibitor, or dalteparin, an LMWH. The trial found that edoxaban was non-inferior to dalteparin for the primary outcome of recurrent VTE or major bleeding during a follow-up period of 12 months (95% confidence interval 0.70 to 1.36; *p* = 0.006) [96]. There was a lower rate of VTE, however non-significant risk difference of −3.4 (−7.0 to 0.2), but the major bleeding rate was significantly higher (risk difference of 2.9 (0.1 to 5.6)) [96]. A non-significant lower VTE rate was seen, but the major bleeding rate was significantly higher in the edoxaban group. Major bleeding events were frequently observed in the subgroup with upper gastrointestinal tract neoplasms [96].

SELECT-D randomized 406 patients with cancer and acute VTE to oral rivaroxaban, a factor, or dalteparin for a treatment duration of 6 months [97]. SELECT-D was a 6-month open-label, pilot, randomized control study that compared rivaroxaban 15 mg BID for three weeks then 20 mg once daily to dalteparin (200 IU/kg once daily for one month, then 150 IU/kg once daily) in patients with VTE and solid or hematologic cancers [97]. The trial found that the VTE recurrence rate was 4% with rivaroxaban and 11% with dalteparin (HR 0.43, 95% CI 0.19 to 0.99) [97]. Major bleeding occurred in 4% with dalteparin and 6% with rivaroxaban (HR 1.83, 95% CI 0.68 to 4.96) and clinically relevant, non-major bleeding occurred in 4% with dalteparin and 13% with rivaroxaban (HR 3.76, 95% 1.63 to 8.69) [97].

CARAVAGGIO was an open label, non-inferiority study that randomized 1170 patients to apixaban (10 mg twice daily for 7 days then 5 mg twice daily) or dalteparin (200 IU/kg once daily for 1 month, then 150 IU/kg once daily) for six months of treatment [98]. The trail resulted in a VTE recurrence rate of 5.6% with apixaban and 7.9% with dalteparin (risk difference –2.3%; HR 0.63, 95% CI 0.37 to 1.07, *p* < 0.001) [98]. Additionally, major bleeding occurred in 3.8% in the apixaban group versus 4% in the dalteparin group (risk difference –0.2%; HR 0.82, 95% CI 0.4 to 1.69, *p* = 0.60) [98].

Given that these oral agents have shown efficacy and provide a more convenient medication dosage form, we have already begun to see a shift in the way PEs are treated in cancer patients [76,79,94,95]. However, since many of these studies have excluded patients with GI tumors or a history of GI bleeding, it is still recommended to continue using LMWH in these patients due to the higher GI bleeding risk seen with DOACs. [76,79,94,95]. If a DOAC is to be utilized, the choice of which oral anticoagulant to be used in a cancer patient should be made on an individual basis with considerations of drug interactions with chemotherapy agents and the type of cancer.

## 12. Special Population: Pregnancy

UFH and LMWH remain the only currently available choices for the safe management of VTE including PE during pregnancy and puerperium. UFH provides the least exposure to the fetus and risk to the mother, since it does not cross the placenta, is not distributed into breast milk, and the dose is easily titrated [99]. LMWHs have a similar safety profile as UFH for the fetus since they also do not cross the placenta. Concerns about distribution into breast milk have been raised [99,100]. Most studies have not demonstrated that the LMWH levels in breast milk are high enough to cause coagulopathy when using standard prophylaxis doses. Further studies are needed for a complete safety profile.

The ability to easily monitor aPPT levels for UFH and anti-Xa levels for LMWHs provides clinicians with the ability to maintain therapeutic concentrations throughout pregnancy. Therapeutic level monitoring provides safety and efficacy for both the fetus and the mother, since changes in the drug volume of distribution and clearance progressively increase throughout pregnancy [22,99,101,102,103]. Additionally, UFH and LMWH have short half-lives, allowing for anticoagulation to be maintained within 24 h of delivery and permitting the safe use of neuroaxial anesthesia if needed [100,104]. Restarting LMWH within six hours post-delivery in patients without bleeding concerns has been shown to be safe. Anticoagulation should be maintained during puerperium [99]. Thromboprophylaxis for future pregnancies with UFH or LMWH is recommended for women at risk [105,106].

The safety profile and pharmacokinetics of DOACs currently do not favor their use during pregnancy or puerperium, since they cross the placenta and distribute into breast milk [99,107]. The use of DOACs may be considered in women who are not or are no longer breast feeding. The inability to readily monitor DOAC levels limits the clinician’s ability to maintain therapeutic levels throughout pregnancy and puerperium due to the pharmacokinetic changes listed above. Maintaining a safe and therapeutic efficacious anticoagulation regimen up to and through the time of delivery requires a multidisciplinary effort and needs to be closely coordinated [99,100,104].

## 13. Special Population: Elderly

The risk of thromboembolic disease, including PE, increases with age [108,109,110]. The trauma to the vascular endothelium caused by a thromboembolic event places the patient at a higher risk of recurrence, requiring prolonged anticoagulation [108,111]. Comorbidities accompanying aging, especially cardiovascular and pulmonary disease, add to the complexity of treatment of thromboembolic disease [112]. The increased risk for bleeding further complicates the safe management of an acute venous thromboembolic event, and prolonged anticoagulation is required to prevent future events [4].

PE in elderly patients may not present with the typical symptoms seen in younger patients, making early diagnosis more challenging in this population. Several studies have found syncope to be the most frequent presenting symptom in elderly patients, versus pleuritic chest pain in a younger population [113,114,115]. Early pharmacotherapy intervention is critical to prevent further thrombus formation, regardless of the patient’s age [108].

Recommendations for initial treatment for PE in a stable patient regardless of the prognosis include DOACs (apixaban or rivaroxaban) or initial LMWH, followed with dabigatran or edoxaban. LMWH or UFH and warfarin may be preferred in patients with reduced renal function (CrCL < 30 mL/min) [116].

Stable patients with a good prognosis may be managed in an outpatient setting. The current recommendations for stable patients with a poor prognosis require hospitalization [116]. A thorough medication history is important prior to starting a DOAC due to the potential drug–drug interactions seen with this class of anticoagulants. P-glycoprotein inhibitors or CYP3A4 inhibitors or inducers should be avoided, since they can alter the plasma of DOACs [116]. The unstable patient requires hospitalization and may be a candidate for thrombolytic therapy, preferably by catheter-directed thrombolysis [116].

## 14. Special Population: COVID-19 Patients

The emergence of severe acute respiratory syndrome coronavirus 2 (SARS-CoV-2) from Wuhan, Hubei province, China, in December 2019 has adversely impacted the world. Amongst the several complications related to SARS-CoV-2 is VTE [117]. A systematic review of 3487 COVID-19 patients from 30 studies demonstrated VTE incidence to be 26%, 12% for PE with or without DVT, and 14% for DVT alone.

The etiology of PE in COVID-19 is secondary to the well-known VTE risk factors, with indirect aspects of the severity of illness and direct effects of SARS-CoV2 viral infection. These patients are at VTE risk due to the recurrent use of intravascular access devices, sedation, and vasopressors, within the intensive care unit (ICU) setting. These factors promote stasis. Respiratory failure, hypoxia, comorbidities, multi-organ failure, obesity, and prolonged immobility are other elements. Additional confounding factors include a history of VTE and or neoplasm, sepsis, surgery, trauma, or stroke [118,119].

COVID-19 is associated with a profound early response of proinflammatory cytokines. This may result in a cytokine storm, increased risk of vascular hyperpermeability, multi-organ failure, and death [120,121]. While thrombin’s primary role is to accelerate clot formation via platelet activation and the conversion of fibrinogen to fibrin; thrombin equally exerts multiple cellular effects. It could further enhance inflammation through proteinase-activated receptors (PARs). Thrombin production is highly regulated via negative feedback mechanisms and physiological anticoagulants, such as the protein C system, antithrombin III, and tissue factor pathway inhibitor [122]. These three control mechanisms may become impaired throughout the inflammatory process, resulting in decreased anticoagulant concentrations secondary to reduced production and increased consumption. This impaired procoagulant–anticoagulant equilibrium results in disseminated intravascular coagulation (DIC), microthrombosis, multi-organ failure, and elevated d-dimer levels [89,123,124]. Hypercoagulable state, endothelial dysfunction, injury, and viral-induced procoagulant effect all also play an immense role in COVID-19-associated PE [125,126,127].

Both LMWH and UFH are recommended for VTE prophylaxis and PE treatment in COVID-19 patients. The recommended dose of the LMWH enoxaparin for VTE prophylaxis is 40 mg (4000 IU) daily. An intermediate dose of LMWH enoxaparin 40 mg subcutaneously every 12 h is recommended in the critically ill, the obese, and those patients with multiple VTE risk factors. A UFH dose of 5000 IU Q8H is recommended for VTE prophylaxis, and doses of up to 7500 IU have been employed in critically ill patients [96,97,98,128,129]. DOACs are recommended in stable patients and in the post-acute phase of PE and outpatient settings when the benefits from their use outweigh the risks. Renal function monitoring with dosage modifications is recommended when LMWH, UFH, and DOACs are employed. Additionally, Anti-Xa monitoring is recommended in patients requiring therapeutic anticoagulation with LMWH, UFH, and DOAC therapy. DOAC plasma level monitoring is also recommended [99,129]. Therapeutic anticoagulation should always be considered first; thrombolytic therapy is recommended in patients who go to develop sub-massive or massive PE. An inferior vena cava filter may be employed in at-risk patients; extracorporeal membrane oxygenator (ECMO) is an option, in conjunction with surgical embolectomy or catheter-directed management [1,100,101]. Several weeks of therapy is recommended with LMWH or DOACs, post-hospitalization for COVID-19 patients [130].

## 15. Conclusions

PE is a medical emergency which affects thousands of American each year. Thousands of Americans die from this condition annually. The therapy for PE has evolved over the years. Traditional therapies such as UFH, VKA, and warfarin are being abandoned by clinicians in favor of LMWH and DOACs. Reversal agents such as 4-factor prothrombin complex concentrate, andaxanet alfa, and the monoclonal antibody idarucizumab have allowed clinicians to push the boundaries of PE management with confidence, particularly in the outpatient setting. Special populations, such as obese, renal dysfunction, liver impairment, cancer, and COVID-19 patients with PE, pose a tremendous therapeutic burden and challenge to clinicians. Despite these challenges, tremendous progress has been made, with demonstrated improved patient outcomes in PE treatment over the last three decades.

## Figures and Tables

**Table 1:** Anticoagulant dosing and special considerations.

Drug	Dose	Special Considerations
UFH ^1^	80 unit/kg IV bolus, followed by an 18-unit/kg/hour infusion [6].	
Enoxaparin	1 mg/kg subQ ^2^ BID ^3^ [12,13].	
Dalteparin	200 IU/kg/day ^4^ subQ for one month, followed by 150 IU/kg/day subcutaneously for months 2 through 6 [14].	Maximum of 18,000 IU per day.
Fondaparinux	<50 kg: 5 mg subQ daily 50–100 mg: 7.5 mg subQ daily >100 kg: 10 mg subQ daily	Initiate warfarin within 72 h and give concomitantly for at least 5 days.
Edoxaban	60 mg po once daily; 30 mg once daily if body weight ≤60 kg	Not for use in patients with CrCl > 95 mL/min ^5^. Dose after 5 to 10 days of initial therapy with a parenteral anticoagulant.
Apixaban	10 mg po twice daily for 7 days followed by 5 mg twice daily [15].	
Rivaroxaban	15 mg po twice daily × 3 weeks, then 20 mg once daily × at least 6 months [16].	Take with food to improve absorption [17,18,19]
Dabigatran	150 mg po BID [20].; 110 mg BID for patients ≥80 years	Dose after 5 to 10 days of initial therapy with a parenteral anticoagulant. Reduce dose to 110 mg BID for patients ≥80 years or ≥75 years with at least one bleeding risk factor.

^1^ UFH: unfractionated heparin; ^2^ subQ: subcutaneously; ^3^ BID: twice a day; ^4^ IU: international units; ^5^ CrCL: creatinine clearance.

**Table 2:** Recommended reversal agents for anticoagulant therapy.

	First Line Reversal Agent	Alternative Reversal Agent(s)
UFH ^1^	Protamine sulfate	
LMWH ^2^	Protamine sulfate	
VKA ^3^	4F-PCC ^4^	FFP ^5^
Dabigatran	Idarucizumab	PCC ^6^ aPCC ^7^
Direct oral factor-Xa inhibitors	Andexanet alfa	PCC aPCC
Fondaparinux	Factor VIIa	aPCC Andexanet alfa

^1^ UFH: Unfractionated heparin; ^2^ LMWH: low molecular weight heparin; ^3^ VKA: vitamin K antagonist; ^4^ 4F-PCC: 4-factor prothrombin complex concentrate; ^5^ FFP: fresh frozen plasma; ^6^ PCC: prothrombin complex concentrate; ^7^ aPCC: activated prothrombin complex concentrate.

**Table 3:** FDA recommendations of DOACs in liver dysfunction.

	Mild Impairment (Child-Pugh Class A)	Moderate Impairment (Child-Pugh Class B)	Severe Impairment (Child-Pugh Class C)
Apixaban	No dosage adjustment.	Use with caution. No dosage adjustments provided.	Use is not recommended.
Rivaroxaban	No dosage adjustment.	Avoid use.	Avoid use.
Edoxaban	No dosage adjustment.	Use is not recommended.	Use is not recommended.
Dabigatran	No dosage adjustment.	Use with caution. No dosage adjustments provided.	No dosage adjustments provided.

## Data Availability

Not applicable.
